# Tuberculosis Incidence Rates during 8 Years of Follow-Up of an Antiretroviral Treatment Cohort in South Africa: Comparison with Rates in the Community

**DOI:** 10.1371/journal.pone.0034156

**Published:** 2012-03-30

**Authors:** Ankur Gupta, Robin Wood, Richard Kaplan, Linda-Gail Bekker, Stephen D. Lawn

**Affiliations:** 1 Department of Clinical Research, Faculty of Infectious and Tropical Diseases, London School of Hygiene and Tropical Medicine, London, United Kingdom; 2 The Desmond Tutu HIV Centre, Institute for Infectious Disease and Molecular Medicine, Faculty of Health Sciences, University of Cape Town, Cape Town, South Africa; National Institute of Allergy and Infectious Diseases, United States of America

## Abstract

**Background:**

Although antiretroviral therapy (ART) is known to be associated with time-dependent reductions in tuberculosis (TB) incidence, the long-term impact of ART on incidence remains imprecisely defined due to limited duration of follow-up and incomplete CD4 cell count recovery in existing studies. We determined TB incidence in a South African ART cohort with up to 8 years of follow-up and stratified rates according to CD4 cell count recovery. We compared these rates with those of HIV-uninfected individuals living in the same community.

**Methodology/Principal Findings:**

Prospectively collected clinical data on patients receiving ART in a community-based cohort in Cape Town were analysed. 1544 patients with a median follow-up of 5.0 years (IQR 2.4–5.8) were included in the analysis. 484 episodes of incident TB (73.6% culture-confirmed) were diagnosed in 424 patients during 6506 person-years (PYs) of follow-up. The TB incidence rate during the first year of ART was 12.4 (95% CI 10.8–14.4) cases/100PYs and decreased to 4.92 (95% CI 3.64–8.62) cases/100PYs between 5 and 8 years of ART. During person-time accrued within CD4 cell strata 0–100, 101–200, 201–300, 301–400, 401–500, 501–700 and ≥700 cells/µL, TB incidence rates (95% CI) were 25.5 (21.6–30.3), 11.2 (9.4–13.5), 7.9 (6.4–9.7), 5.0 (3.9–6.6), 5.1 (3.8–6.8), 4.1 (3.1–5.4) and 2.7 (1.7–4.5) cases/100PYs, respectively. Overall, 75% (95% CI 70.9–78.8) of TB episodes were recurrent cases. Updated CD4 cell count and viral load measurements were independently associated with long-term TB risk. TB rates during person-time accrued in the highest CD4 cell count stratum (>700 cells/µL) were 4.4-fold higher that the rate in HIV uninfected individuals living in the same community (2.7 versus 0.62 cases/100PYs; 95%CI 0.58–0.65).

**Conclusions/Significance:**

TB rates during long-term ART remained substantially greater than rates in the local HIV uninfected populations regardless of duration of ART or attainment of CD4 cell counts exceeding 700 cells/µL.

## Introduction

As the 2015 end-point for the Millennium Development Goals approaches, HIV-associated tuberculosis (TB) remains a substantial challenge to global health, accounting for 13% (1.1 million) of new TB cases and approximately 25% of global deaths from HIV/AIDS [1]. The greatest burden of disease is in the countries of sub-Saharan Africa where approximately 80% of cases occur. In addition to the DOTS TB control strategy, interventions to address HIV-associated TB include isoniazid preventative therapy (IPT), infection control, intensive case finding and antiretroviral therapy (ART) [2]–[4]. However, ART remains the only TB preventative intervention implemented on a large scale. By the end of 2009 treatment had been provided for 3.91 million people in sub-Saharan Africa and to 0.97 million people in South Africa alone [5].

A meta-analysis of observational cohort studies reported a 67% (95% CI 61–73) reduction in TB risk in patients receiving ART, indicating its key role in prevention of HIV-associated TB [2], [6]. High initial rates of TB in the first few months of ART rapidly decrease with time-dependent reductions during the first 2–3 years of treatment [7]–[15]. However, these data are limited. Median follow-up is generally short and infrequently exceeds 2 years. Calculation of long-term TB rates has therefore been based on small numbers of participants and events, as reflected by wide confidence intervals. Many patients included in these studies have not attained their greatest potential CD4 cell count recovery, and much person-time accrued is therefore associated with lower CD4 cell counts. Moreover, cases are often not microbiologically proven. Thus, true long-term TB rates and risk factors remain imprecisely defined.

Data on the incidence of TB during long-term ART and comparisons with local HIV uninfected populations are important for providing much needed insight into the likely long-term impact of ART on the HIV-associated TB epidemic at a population level. Such data are also needed to inform the implementation of additional preventive interventions. However, previously low rates of HIV testing among TB patients in many settings has meant that it has not been possible to accurately estimate the TB notification rate in the HIV-uninfected sub-population in the relevant local communities. In this study, we have substantially built on existing findings in a large ART cohort in Cape Town, analysing data from patients treated for up to 8 years and from patients who attained very high levels of CD4 cell count recovery. We provide a careful comparison of these rates with those measured among HIV-uninfected adults living in the same local community, which have not previously been available.

## Methods

### Study setting

This study was part of ongoing research at the Gugulethu ART service in the Klipfontein sub-district of Cape Town, which has been previously well described [16]. It has a predominantly African population of over 300,000 who mostly live in low socioeconomic conditions. At the time of programme commencement, antenatal HIV seroprevalence was approximately 30% and the annual TB notification rate was estimated to be greater than 1,500/100,000 [16]–[18].

Referrals were received from primary care clinics, antenatal clinics and TB clinics. Programmatic criteria for starting ART followed national guidelines, based on the 2002 WHO recommendations, providing free ART for those with WHO stage 4 disease or a CD4 cell count <200 cells/µL [19]. The first-line ART regimen comprised of two nucleoside reverse transcriptase inhibitors and a non-nucleoside reverse transcriptase inhibitor. The second-line regimen included protease inhibitors. Patients did not receive IPT as per national practice at that time.

Patients received routine clinical review at a screening visit, treatment initiation, after 4, 8 and 16 weeks of ART, and thereafter at least every 4 months. Patients had open access to the clinic at all other times. Blood CD4 cell counts and plasma viral load levels were done routinely at baseline and every four months.

### Tuberculosis screening and diagnosis

TB screening was conducted at baseline using a symptom-screening questionnaire to identify patients who required further TB investigations. Investigations available to diagnose TB at baseline or during ART included sputum induction, sputum smear fluorescence microscopy, automated liquid culture of sputum using mycobacterial growth indicator tubes (MGIT 960, Becton Dickinson, Sparks, Maryland, USA), chest radiology, ultrasonography and fine needle lymph node aspiration and cytology. All microbiological specimens were analysed in accredited laboratories, and all positive cultures were speciated by polymerase chain reaction. TB treatment was rifampicin-based and under a directly observed treatment short-course (DOTS) strategy provided through a network of community clinics.

‘Incident TB’ was defined as a new clinical episode of TB diagnosed after initiation of ART irrespective of date of symptom onset. Diagnoses of ‘recurrent TB’ were made with reference to any previous episodes of TB, including those made prior to ART enrolment. All TB diagnoses fulfilled WHO criteria for resource-constrained settings with high HIV prevalence [20]. Smear-negative pulmonary TB, in addition to two sputum samples being negative for acid-fast bacilli (AFB), was defined by radiological abnormalities consistent with active TB, and either a positive culture for *Mycobacterium tuberculosis* complex or a clinician's decision to treat with a full course of anti-tuberculosis chemotherapy. Extrapulmonary TB was either culture or smear-positive from an extrapulmonary site, or had strong histological or clinical evidence of active TB with a clinician's decision to treat with a full course of anti-tuberculosis chemotherapy. Patients with both pulmonary and extrapulmonary disease were classified as extrapulmonary TB. For diagnoses of recurrent episodes of incident TB during ART, patients must have completed a full course of anti-tuberculosis treatment with resolution of symptoms.

### Data collection and ethics

Data were collected from patients enrolled consecutively into the ART programme from September 2002 until May 2006. Patients were excluded if they did not initiate ART, were non-naïve to ART at enrolment due to transfer in from another service, and if aged less than 16 years. Information on TB events was obtained from prospectively maintained structured clinical records. Laboratory results were transferred weekly to an electronic database. All patients enrolled in the study gave written, informed consent to participate. The collection of data on this study cohort and use of data from the Cape Town TB register for research purposes was approved by the Research Ethics Committee of the University of Cape Town.

### TB rates in the community

Community TB services were provided through the same local clinics as for the study cohort, supported by a comprehensive laboratory service. TB cases were recorded in an electronic TB register as per South African national guidelines [21]. TB notification data, including the HIV status of diagnosed cases, for the local population (Klipfontein sub-district) was extracted from the Cape Town electronic TB register. Data corresponding to 2009 were used to calculate community rates as HIV status had been determined for a high proportion (87%) of the TB notifications in this year, which was also during the latter part of the overall follow-up period by which time good immune recovery would have occurred in patients recruited in earlier years. Population and HIV prevalence data stratified by age, sex and HIV status were obtained from the National Department of Health/Health Information System Program and the Actuarial Society of South Africa AIDS demographic model, respectively [22]–[24]. These data were used to provide the necessary numerators and denominators to calculate TB rates stratified by HIV-status, age and gender as previously described [25].

### Data analysis

Data were analysed using Stata version 11.0 (College Station, Texas, USA). Person-time at risk of TB was accrued from the date of starting ART until death, transfer, loss to follow-up (LTFU) or censoring of observations on 1^st^ January 2011. Any patient over 12 weeks late for a scheduled appointment who failed to return to the ART programme and could not be located by community-based follow-up was classified as LTFU. Person-time accrued during treatment of prevalent or incident TB was excluded when calculating TB incidence rates, and all episodes of incident TB were included in the analysis. Chi-squared tests were used for comparing proportions, t-tests for comparing means and Wilcoxon rank-sum tests for comparing medians. All statistical tests were two-sided at α value of 0.05.

Person-time was divided into intervals predefined by the CD4 count measurements done at least every 16 weeks. Each interval was defined by the CD4 count and viral load at the start of the interval, and categorised into CD4 cell count strata and viral load strata. If CD4 cell count or viral load measurements were missing for one or more intervals, a mean of the preceding and subsequent values were used (1.6% of all intervals). Poisson regression models were used to calculate incidence rate ratios (IRR) with 95% confidence intervals (CI) for time-updated and baseline characteristics. Random effects modelling was used to account for clustering of person-time for individuals with more than one episode of incident TB. A multivariate model was constructed using Poisson regression and based on forward selection; *a priori* risk factors (age and gender) and variables identified as being associated with incident TB during the univariate analysis were included. Likelihood ratio tests were used to investigate interactions between variables, statistical hypotheses and linear trends. TB incidence rates in the local community were calculated using all TB notifications during 2009 and mid-year population denominators from the same year stratified using HIV prevalence data.

## Results

### Cohort characteristics

**Figure 1:**
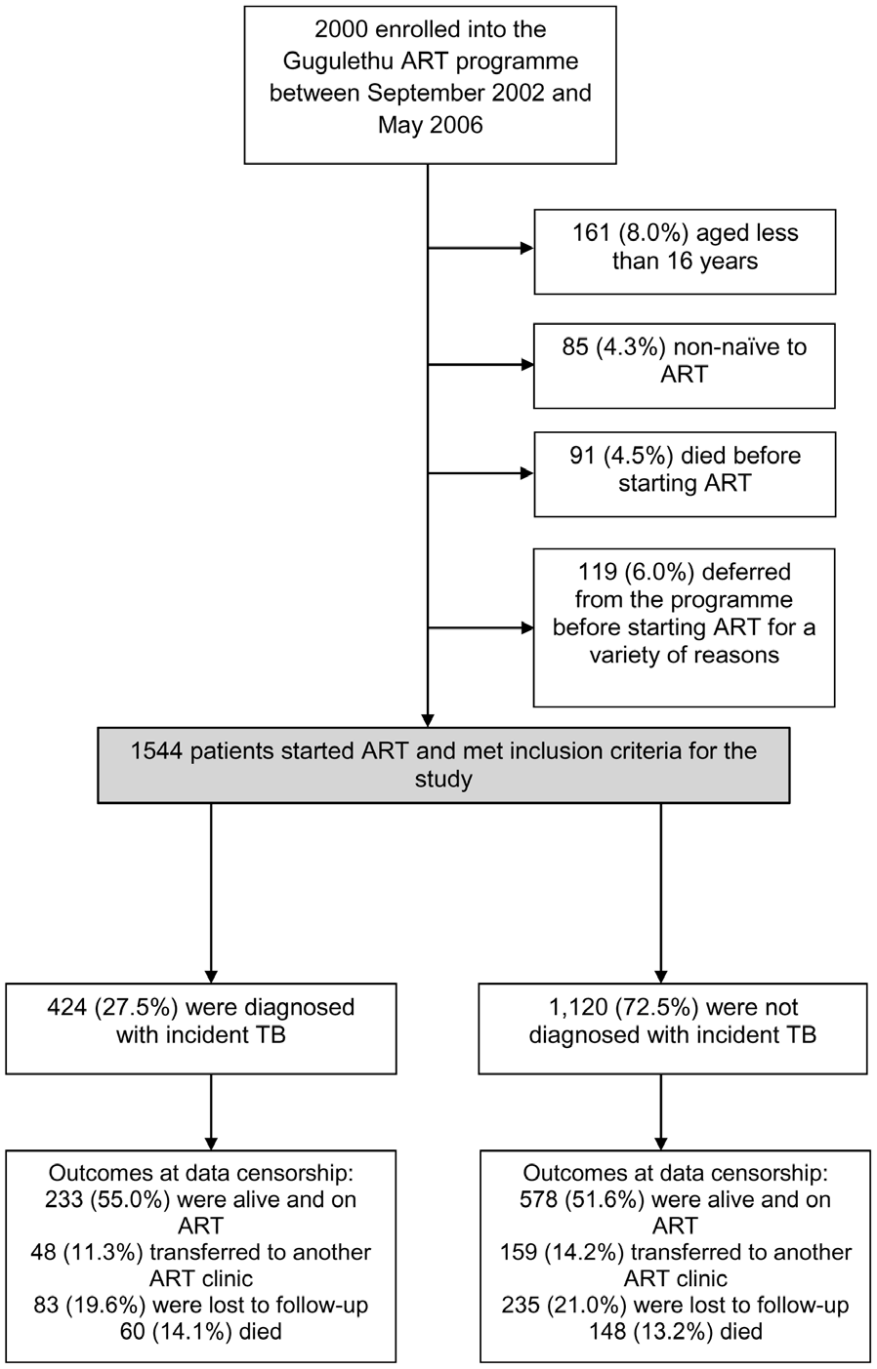
Patient enrolment and outcomes during follow-up.

Of 2000 patients enrolled into the ART programme between September 2002 and May 2006, a total of 456 were excluded because they were aged less than 16 years (n = 161), non-naïve to ART (n = 85), died before starting ART (n = 91) or deferred from starting ART for a variety of reasons (n = 119). 1544 (77.2%) initiated ART and were included in the analysis (**Figure 1**). Of these patients, 208 (13.5%) died during ART, 318 (20.6%) were LTFU and 207 (13.4%) were transferred to other ART services. Median follow-up time for patients included in the analysis was 59.4 months (IQR 29.0–69.7; range 0.0–98.8), and a total of 6506 person-years (PYs) of follow-up were accrued after excluding time on TB treatment.

The mean age of the cohort was 34 years, and the majority of patients were women (70.1%). Overall, patients had advanced immunodeficiency at enrolment with a median baseline CD4 cell count of 98 cells/µL (IQR 48–155) (**Table 1**). Almost half of patients (47.0%) had a past history of TB and had completed a course of anti-tuberculosis treatment before starting ART. Approximately one third (30.0%) of patients were receiving anti-tuberculosis treatment (prevalent TB) at the time of ART initiation. Characteristics did not differ for those alive and retained in the cohort at data censorship and those transferred out or LTFU, except those retained in the cohort had an older mean age.

**Table 1:** Patient characteristics at initiation of antiretroviral therapy (ART).

		Overall n = 1544	No incident TB n = 1120	Incident TB n = 424	p-value
**Mean age (years)**		34.0	34.3	33.2	0.020
**Gender**	**Male**	462 (29.9)	326 (29.1)	136 (32.1)	
	**Female**	1,082 (70.1)	794 (70.9)	288 (67.9)	0.256
**WHO stage**	**1&2**	305 (19.8)	237 (21.2)	68 (15.1)	
	**3**	861 (55.8)	625 (55.8)	236 (55.6)	0.049
	**4**	378 (24.5)	258 (23.0)	120 (28.3)	
**Baseline CD4 cell count (cells/µL)** a	**Median [IQR]**	98 [48–155]	100 [47–157]	94 [48–149]	0.442
	**<50**	375 (26.0)	272 (26.3)	103 (25.3)	
	**50–99**	349 (24.2)	241 (23.3)	108 (26.5)	
	**100–149**	321 (22.3)	225 (21.8)	96 (23.6)	0.314
	**≥150**	396 (27.5)	296 (28.6)	100 (24.6)	
**Baseline viral load (log_10_ copies/ml)** b	**Median [IQR]**	4.87 [4.46–5.26]	4.86 [4.43–5.67]	4.88 [4.51–5.25]	0.598
	**<5**	838 (58.7)	602 (58.7)	236 (58.7)	
	**≥5**	590 (41.3)	424 (41.3)	166 (41.3)	0.991
**Past history of TB** c	**No**	819 (53.0)	632 (56.4)	187 (44.1)	
	**Yes**	725 (47.0)	488 (43.6)	237 (55.9)	<0.001
**Prevalent TB** d	**No**	1,080 (70.0)	757 (67.6)	323 (76.2)	
	**Yes**	464 (30.0)	363 (32.4)	101 (23.8)	0.001

Data are numbers (%) unless otherwise stated. ART, antiretroviral therapy; TB, tuberculosis; IQR, interquartile range.

a Data missing for 103 patients.

b data missing for 116 patients.

c includes all TB episodes where anti-TB treatment was completed prior to ART enrolment.

d is TB episodes where patients are taking anti-TB treatment at ART initiation.

### TB diagnoses

Incident episodes of TB occurred among 424 (27.5%) patients during ART. In total there were 484 episodes of incident TB, with 49 patients having 2 episodes and 5 patients having greater than 2 episodes of incident TB. For episodes where disease site was recorded (98.8%), 362 (74.8%) were pulmonary TB and 116 (24.0%) were extrapulmonary TB. Overall, 73.6% (95% CI 69.4–77.4) of TB episodes were confirmed by culture. Baseline characteristics of those who were and were not diagnosed with incident TB were similar (**Table 1**), except those with incident TB were younger (p = 0.02), more likely to have had a history of TB before ART initiation (p<0.001) and less likely to have been receiving treatment for prevalent TB at the time of ART initiation (p = 0.001). By data censorship, the cumulative proportion of patients who had ever had a TB diagnosis (before or during ART) was 75.4% (73.2–77.6).

### TB incidence rates over time

The overall TB incidence rate during ART was 7.44 cases/100 PYs (95% CI 6.80–8.13). TB rates were highest in the first year of ART at 12.43 cases/100 PYs (95% CI 10.82–14.35) and decreased by almost half in the second year to 6.71 cases/100 PYs (95% CI 5.48–8.30) (**Table 2**). Thereafter, rates decreased to 4.92 (95% CI 3.64–8.62) cases/100 PYs beyond five years of ART. 75.0% (95% CI 70.9–78.8%) of TB burden was recurrent disease, but rates of new and recurrent TB did not differ significantly (6.50 and 7.81 cases/100 PY respectively; IRR 1.20; 95% CI 0.96–1.50; p = 0.098). Rates calculated using only culture-proven TB episodes did not differ significantly from the overall rates. Culture-confirmed TB rates during the first, second, third, fourth, fifth and beyond five years of ART were 9.86 (IRR 0.79; 95% CI 0.63–1.00), 4.49 (IRR 0.71; 95% CI 0.5–1.01), 4.39 (IRR 0.71; 95% CI 0.49–1.04), 5.57 (IRR 0.83; 95% CI 0.58–1.21), 4.12 (IRR 0.76; 95% CI 0.48–1.18) and 4.16 (IRR 0.85; 95% CI 0.52–1.38) cases/100 PYs respectively.

**Table 2:** Total, new and recurrent tuberculosis (TB) incidence rates according to duration of antiretroviral therapy.

ART Duration(month)	Total TB episodes	PY at risk	Rate (per 100 PY) [95% CI]	New^a^ TB episodes	PY at risk	Rate (per 100 PY) [95% CI]	Recurrent TB^b^ episodes	PY at risk	Rate (per 100 PY) [95% CI]	% recurrent TB [95% CI]
**0–12**	174	1400	**12.43** [10.82–14.35]	46	438	10.49 [7.88–14.26]	128	962	13.31 [11.33–15.74]	73.6 [66.4–79.9]
**12–24**	84	1252	**6.71** [5.48–8.30]	29	375	7.74 [5.44–11.38]	55	878	6.27 [4.88–15.74]	65.5 [54.3–75.5]
**24–36**	70	1138	**6.15** [4.90–7.82]	15	323	4.65 [2.86–8.08]	55	816	6.75 [5.23–8.86]	78.6 [67.1–87.5]
**36–48**	68	1023	**6.64** [5.30–8.44]	15	282	5.32 [3.25–9.29]	53	741	7.15 [5.55–9.38]	77.9 [66.2–87.1]
**48–60**	49	899	**5.45** [4.18–7.25]	7	242	2.89 [1.41–6.90]	42	657	6.39 [4.81–8.70]	85.7 [72.8–94.1]
**>60**	39	793	**4.92** [3.64–8.62]	9	202	4.45 [2.37–9.33]	30	591	5.08 [3.59–7.42]	76.9 [60.7–88.9]
**Overall**	484	6506	**7.44** [6.82–8.13]	121	1862	6.50 [5.41–7.84]	363	4644	7.81 [7.06–8.68]	75.0 [70.9–78.8]

ART, antiretroviral therapy; TB, tuberculosis; PY, person-years; CI, confidence intervals.

a no previous episodes of TB, a time-updated variable.

b any previous episodes of TB, including prior to ART enrolment, a time-updated variable.

### TB incidence rates and updated CD4 cell counts

**Figure 2:**
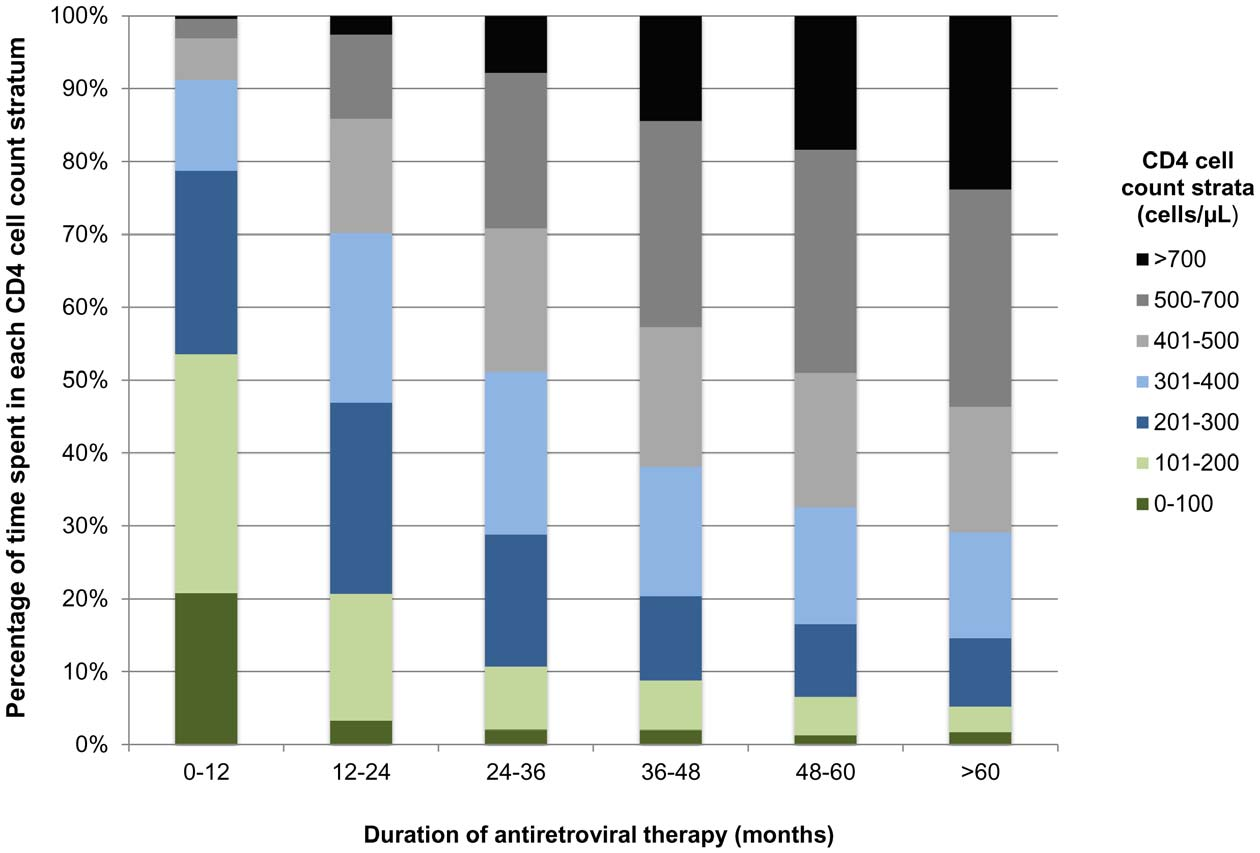
Proportions of person-time accrued in updated CD4 cell count strata by duration of antiretroviral therapy (months) for the whole study cohort.

Person-time accrued within different updated CD4 cell count strata was calculated (**Figure 2**). During the first year of ART patients accrued more than 50% of time at CD4 cell counts <200 cells/µL, compared to less than 5% of time beyond the fifth year of ART. Time accrued at higher CD4 cell counts increased each year after ART initiation, and over 50% of time accrued beyond the fourth year of ART was spent at CD4 cell counts >500 cells/µL.

TB incidence rates were then calculated by updated CD4 count strata (**Table 3**). Rates were highest during person-time accrued with CD4 cells counts <100 cells/µL at 25.49 cases/100 PYs (95% CI 21.57–30.33), decreasing to 2.70 cases/100 PYs (95% CI 1.73–4.47) during person-time accrued in the highest CD4 cell count category (>700 cells/µL). The median CD4 count in the >700 cells/µL stratum was 821 cells/µL.

**Table 3:** Total, new and recurrent tuberculosis (TB) incidence rates by updated CD4 cell count strata.

Updated CD4 cell count strata (cells/µL)	Total TB episodes	PY at risk	Rate (per 100 PY)[95% CI]	New^a^ TB episodes	PY at risk	Rate (per 100 PY) [95% CI]	Recurrent TB^b^ episodes	PY at risk	Rate (per 100 PY) [95% CI]	% recurrent TB [95% CI]
**≤100**	111	436	**25.49** [21.57–30.33]	27	93	29.14 [19.87–43.97]	84	343	24.50 [20.25–29.94]	75.7 [66.9–82.7]
**101–200**	110	979	**11.23** [9.42–13.51]	35	292	11.99 [8.64–17.03]	75	687	10.91 [8.80–13.69]	68.2 [59.0–76.1]
**201–300**	92	1171	**7.86** [6.44–9.68]	20	343	5.83 [3.81–9.35]	72	828	8.70 [6.96–11.03]	78.3 [68.8–85.5]
**301–400**	57	1137	**5.01** [3.89–6.56]	15	351	4.27 [2.61–7.44]	42	786	5.34 [3.98–7.35]	73.7 [61.0–83.4]
**401–500**	49	969	**5.06** [3.84–6.80]	12	281	4.27 [2.48–7.99]	37	688	5.38 [3.94–7.54]	75.5 [61.9–85.4]
**501–700**	48	1184	**4.05** [3.10–5.42]	9	354	2.54 [1.36–5.34]	39	830	4.70 [3.48–6.49]	81.3 [68.1–89.8]
**>700**	17	630	**2.70** [1.73–4.47]	3	148	2.02 [0.63–9.74]	14	482	2.91 [1.78–5.09]	82.4 [59.0–93.8]

ART, antiretroviral therapy; TB, tuberculosis; PY, person-years; CI, confidence intervals.

a no previous episodes of TB, a time-updated variable.

b any previous episodes of TB, including prior to ART enrolment, a time-updated variable.

### Risk factors for incident TB

In the crude analysis (**Table 4**) updated CD4 cell counts showed the strongest association with incident TB (p<0.001). TB incidence was 9 times greater during person-time accrued at CD4 cell counts <100 cells/µL compared person-time at CD4 cell counts >700 cells/µL (IRR 9.45; 95% CI 5.78–15.42; p<0.001). Viral loads >1000 copies/ml were associated with over three times the risk of incident TB (IRR 3.34; 95% CI 2.78–4.01; p<0.001). Duration of ART was also associated with incident TB in crude analyses. Patients receiving ART for over 5 years were 60% less likely to be diagnosed with TB than during the first year of ART (IRR 0.40; 95% CI 0.28–0.55; p<0.001). Of the baseline characteristics, WHO stage at starting ART was associated with increased risk of incident TB (IRR 1.64; 95% CI 1.24–2.15; p = 0.002).

**Table 4:** Univariate and multivariate analyses of risk factors for incident tuberculosis (TB) during antiretroviral therapy.

		Crude analysis			Multivariate analysis^f^		
		IRR	95% CI	p-value	IRR	95% CI	p-value
**Age at enrolment** a		0.99	0.98–1.00	0.081	0.98	0.97–1.00	0.008
**Gender**	**M**	1			1		
	**F**	1.09	0.91–1.31	0.357	0.98	0.80–1.19	0.860
**WHO stage at enrolment**	**1 & 2**	1			1		
	**3**	1.35	1.06–1.74	0. 002	1.26	0.97–1.65	0.018
	**4**	1.64	1.24–2.15		1.56	1.15–2.10	
**Baseline CD4 cell count (cells/µL)** b	**<50**	1					
	**50–99**	0.98	0.76–1.25				
	**100–149**	1.01	0.78–1.31	0.258			
	**≥150**	0.81	0.63–1.04				
**Baseline viral load (log_10_ copies/ml)** c	**<5**	1					
	**≥5**	1.03	0.85–1.23	0.780			
**Duration of ART (months)**	**0–12**	1			1		
	**12–24**	0.54	0.42–0.70		1.01	0.76–1.33	
	**24–36**	0.49	0.38–0.65		1.08	0.79–1.46	
	**36–48**	0.53	0.41–0.70	<0.001	1.25	0.92–1.69	0.812
	**48–60**	0.44	0.32–0.60		1.10	0.79–1.55	
	**>60**	0.40	0.28–0.55		1.05	0.73–1.52	
**Updated CD4 cell count (cells/µL)** d	**>700**	1			1		
	**501–700**	1.50	0.89–2.53		1.56	0.92–2.63	
	**401–500**	1.87	1.10–3.21		1.96	1.14–3.39	
	**301–400**	1.86	1.10–3.14	<0.001	1.96	1.16–3.33	<0.001
	**201–300**	2.91	1.76–4.83		2.97	1.75–5.01	
	**101–200**	4.16	2.53–6.85		3.81	2.24–6.46	
	**0–100**	9.45	5.78–15.42		6.94	3.96–12.16	
**Updated viral load (copies/ml)** d	**<1000**	1			1		
	**≥1000**	3.34	2.78–4.01	<0.001	1.74	1.39–2.17	<0.001
**Recurrent TB** e	**No**	1			1		
	**Yes**	1.20	0.96–1.50	0.098	1.04	0.82–1.31	0.763

ART, antiretroviral therapy; TB, tuberculosis; IRR, incident rate ratio; CI, confidence interval. All p-values were calculated using the likelihood ratio test. There were no interactions between variables in the multivariate analysis.

a Age is included as continuous variable, the IRR represents a 1 year increase in age.

b Measurement taken at ART enrolment, data missing for 103 patients.

c Measurement taken at ART enrolment, data missing for 116 patients.

d CD4 cell counts and viral load are time-updated variables using serial measurements taken during ART.

e Any previous episodes of TB, including prior to ART enrolment, a time-updated variable.

f data for all 1544 patients included in final model.

In a multivariate Poisson regression model, the updated CD4 cell count was the variable most strongly associated with the development of incident TB during ART (**Table 4**). TB incidence was nearly 7 times greater during person-time accrued at CD4 cell counts <100 cells/µL compared person-time at CD4 cell counts >700 cells/µL (IRR 6.94; 95% CI 3.96–12.16; p<0.001). Detection of a viral load ≥1000 copies/ml was also independently associated with incident TB (IRR 1.74; 95% CI 1.39–2.17; p<0.001) whereas duration of ART was not. Age and WHO stage at ART enrolment were also independently associated with development of incident TB during ART.

### Comparison with community TB rates

**Figure 3:**
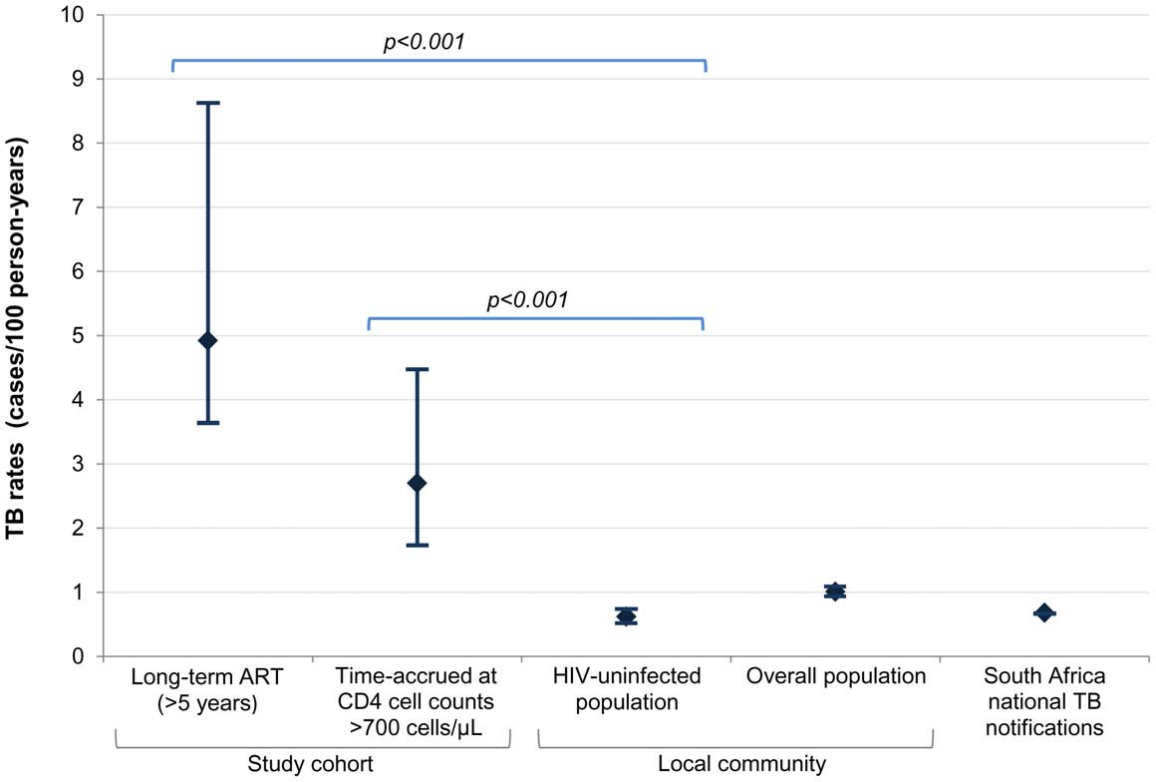
Graph comparing tuberculosis (TB) rates and 95% confidence intervals for the study cohort, local population and South Africa national notification rates. TB rates (cases/100 person-years, [95% CI]) for patients on long-term ART >5 years (4.92, [3.64–8.62]), during person-time accrued with updated CD4 cell counts >700 cells/µL (2.70, [1.73–4.47]), the HIV uninfected population (0.62, [0.58–0.65]) and overall population (1.01, [0.97–1.04) of the local community in 2009, and the South Africa national TB notification rates in 2009 (0.68, [0.678–0.682]).

In the local community 2922 TB episodes were notified during 289,304 PYs, giving an overall TB rate of 1.01 cases/100 PYs (95% CI 0.97–1.04). The HIV uninfected population had 1262 TB episodes during an estimated 205,117 PYs, a rate of 0.62 cases/100 PYs (95% CI 0.58–0.65), less than 25% of the rate in HIV-infected patients during person-time accrued at CD4 cell counts >700 cells/µL (**Figure 3**). The proportion of cases in the study cohort who were female was substantially greater than the proportion in the community (70.1% versus 31.3%, respectively; p<0.001), reflecting the typical ART cohort composition in sub-Saharan Africa. Therefore TB incidence rates stratified by gender and age were calculated and compared with ART patients with similar characteristics in the study cohort and with updated CD4 counts >700 cells/µL. TB rates were 7 times higher in HIV-infected women receiving ART aged 16–44 years compared to uninfected women (2.70 and 0.37 cases/100PYs respectively; IRR 7.32; p<0.001) and 4 times higher in HIV-infected men receiving ART aged 16–44 years compared to uninfected men in the same local community (3.31 and 0.82 cases/100PYs respectively; IRR 4.01; p = 0.024).

## Discussion

The large size of this cohort, prolonged follow-up (median of 5.0 years), high rates of microbiological diagnosis and frequent CD4 count and viral load measurements allowed us to calculate with greater precision than existing studies how long-term TB incidence rates vary according to duration of ART and degree of CD4 cell count recovery. Beyond the first year of ART, TB incidence rates remained fairly constant, ranging between 6.71 and 4.92 cases/100PYs, and TB risk was not associated with increasing duration of ART in multivariate analysis. Instead, these data very clearly confirm that TB risk over time is very strongly related to the immunovirological response to ART. However, even when patients' CD4 cell counts exceeded a threshold of 700 cells/µL and reached a median of 821 cells/µL, the TB incidence rate remained more than 4-fold higher than the rate among HIV-uninfected individuals living in the same community. These data provide strong confirmatory evidence that long-term ART is not associated with normalisation of TB risk even in those with excellent CD4 cell count responses.

Several studies of TB incidence during ART have reported that rates stabilise above the estimated background population rate [12]–[14]. However, these analyses have lacked the appropriate comparisons with rates among HIV-uninfected individuals of similar age and gender living in the same community. In Cape Town, introduction of provider-initiated testing and counselling in 2005 has resulted in dramatic increases in rates of HIV-testing among TB patients [26]. For the first time, use of TB notification data, patient HIV status and population denominators has made it possible to derive estimates of TB incidence rates among the HIV-infected and uninfected sub-populations in the city and by sub-district [25]. In all comparisons of TB incidence rates stratified by HIV-status, gender and age in this study, rates among HIV-infected individuals attaining CD4 cell counts >700 cells/µL during ART remained between 4 and 7-fold higher than the HIV-uninfected comparison groups.

These data confirm that TB incidence rates even after 5 years of ART remain high (approximately 5% per year) and a key explanation for this was that approximately half the person-time between 5 and 8 years of follow-up was accrued at CD4 cell counts <500 cells/µL. The observation that rates even among those who achieved CD4 cell counts >700 cells/µL remained much higher than background is consistent with the hypothesis that ART is insufficient to fully restore immune responses to *Mycobacterium tuberculosis* [27]. This may be due to functional immune defects that may persist despite excellent CD4 cell count recovery [27]. Another possibility that we cannot rule out is that individuals living with HIV in this community have greater exposure to TB or other common risk factors for the acquisition of both infections. However, in communities such as this one with generalised HIV and TB epidemics, this is a less likely explanation compared with high income settings where epidemics are highly concentrated in specific population sub-groups. We doubt that nosocomial TB transmission is an important factor among healthy patients on ART with CD4 cell counts >700 cells/µL, as such patients spend relatively little time in health care settings and prevailing TB transmission in the community is so high [28], [29]. Further research is warranted to investigate this excess TB risk despite good response to ART.

This study also calculated rates of new and recurrent TB, although we were unable to distinguish between recurrence due to reinfection and endogenous reactivation of the original strain (relapse). Studies in similar high-burden TB settings have attributed most incident TB episodes to reinfection in the context of HIV through molecular typing of mycobacterial isolates [15], [30], [31]. Although recurrent TB was not associated with increased TB risk in the multivariate analysis, high rates of recurrent disease reflect high TB exposure and force of infection this setting [32]. Recurrent disease accounted for 75% of the burden of incident TB in this study, compared to 26% of the overall and 43% of HIV-infected populations in Cape Town in 2009 [25]. This high proportion of recurrent disease may be explained by the fact that TB is a key reason for initial presentation to the health services and HIV diagnosis. Subsequent prolonged survival during ART with persisting high TB risk means that much of the subsequent incident TB is therefore recurrent.

Lack of viral load suppression was independently associated with a higher risk of incident TB in this study. This association has been noted in both developing and developed countries. Incident TB during ART has also been associated with virological failure in sub-Saharan African ART cohorts [33], [34]. In vitro research has shown HIV itself can cause functional impairment of anti-TB immune response independently of loss CD4 cells [35]. However, the temporal relationship between a detectable viral load during ART and incident TB is less clearly defined.

The data from this study suggest that the greatly expanding number of patients receiving long-term ART in high TB burden settings represents a population with long-term heightened susceptibility to TB. With an estimated 1 million people receiving ART in South Africa in 2009, these patients would develop approximately 50,000 episodes of TB each year long-term, and this number will grow each year with ongoing ART scale-up [5]. This is a significant burden of disease that is a direct result of the major increases in survival that are associated with ART. This observation supports the conclusion that ART scale-up is likely to have more limited impact on control of the HIV-associated TB epidemic than might have been concluded from cohort studies of the short-term impact of ART [2].

A number of interventions are needed to reduce the huge burden of TB that we have described. Starting ART at higher CD4 cell counts would reduce the person-time accrued at low CD4 cell counts during which patients remain at high TB risk [2]. This, together with interventions to improve ART outcomes, including improved adherence and reduced losses to follow-up, will help increase ART's impact on HIV-associated TB [2]. Second-line ART should be offered to patients with virological failure as it is associated with improved immunological outcomes, and hence a reduction in incident TB risk [36]–[38]. Intensified case finding and implementation of other infection control interventions are needed to reduce the risk of nosocomial TB transmission [39]. Intensified screening at baseline expedites TB diagnosis and is associated with substantial reductions in TB incidence rates during the initial months of ART [40]. Serial screening at intervals during ART might have additional impact._ENREF_49 Novel diagnostics, such as the Xpert MTB/RIF assay and Determine TB-LAM urine lipoarabinomannan antigen point-of-care assay now provide the means for rapid screening of individuals within the health care setting [41], [42]. IPT and ART are interventions that work via complementary mechanisms in the prevention of HIV-associated TB [43]. Observational data show an additive effect of IPT when used concurrently with ART, although randomised control trials addressing this question directly are also underway such as one in South Africa (http://clinicaltrials.gov/ct2/show/NCT00463086) [43]–[46].

The observational design of this study has inherent limitations, but may also improve generalizability as our programmatic conditions will be similar to other ART clinics. However, the HIV-infected cohort potentially had better access to healthcare services compared to the HIV-uninfected population and therefore there may have been differences in case ascertainment between the two. There were some biological and social risk factors we were unable to account for in this study, for example diabetes and smoking. These factors alone are unlikely to account for the discrepancy in TB rates between the HIV-uninfected population and persons reaching the highest CD4 cell counts. Although high, our rates of non-death losses are similar to other South African studies, and probably reflect the highly mobile population of the Cape townships [47], [48]. Anti-TB treatment completion could not be confirmed for TB episodes treated prior to ART programme enrolment, but any inadequately treated TB is likely to have been diagnosed during pre-ART screening. In the absence of post-mortem studies, we were unable to determine the proportion of deaths that were associated with unascertained TB.

Strengths of this study include over twice the median follow-up of any study of comparable size assessing TB incidence during ART. The baseline characteristics, immune recovery and outcomes in this cohort were comparable to other ART cohorts from sub-Saharan Africa [7]–[10], [49]. Although data were from a single site in a community with TB rates that are likely to be higher than communities elsewhere in sub-Saharan Africa, the relationship between incident TB, response to ART and comparisons to background rates that we have carefully documented are likely to be applicable to other settings. Over 73% of cases were microbiologically proven and availability of routine mycobacterial culture greatly enhanced the reliability of TB diagnoses. Routine monitoring of CD4 cell count and viral load every four months provided accurate information on time-updated immunovirological responses. The availability of an electronic TB register, high rates of HIV-testing in TB patients in Cape Town and use of population denominators allowed us to calculate TB rates specific to the sub-district where the ART clinic was based.

In conclusion, TB incidence rates during long-term ART remained substantially greater than rates in the local HIV uninfected populations regardless of prolonged duration of ART or attainment of CD4 cell counts exceeding 700 cells/µL. With ongoing rapid expansion of ART, TB developing in patients receiving long-term ART will represent an increasing burden of disease that requires additional interventions to control.
